# The effect of cyclic loading on the fracture resistance of 3D-printed and CAD/CAM milled zirconia crowns—an in vitro study

**DOI:** 10.1007/s00784-023-05229-2

**Published:** 2023-08-24

**Authors:** Ashraf Refaie, Christoph Bourauel, Ahmed Mahmoud Fouda, Ludger Keilig, Lamia Singer

**Affiliations:** 1 Oral Technology, Dental School, Medical Faculty, University Hospital of Bonn, Welschnonnenstr. 17, 53111 Bonn, North Rhine-Westphalia Germany; 2 Department of Fixed Prosthodontics, Faculty of Dentistry, Fayoum University, Faiyum, Egypt; 3 Suez Canal University, Ismailia, Egypt; 4 Department of Prosthodontics, Preclinical Education and Dental Materials Science, Dental School, Medical Faculty, University Hospital of Bonn, 53111 Bonn, North Rhine-Westphalia Germany; 5 Department of Orthodontic, Dental School, Medical Faculty, University Hospital of Bonn, 53111 Bonn, North Rhine-Westphalia Germany

**Keywords:** 3D printing, Zirconia, Fracture resistance, Cyclic loading

## Abstract

**Objectives:**

The aim of this study was to evaluate the effect of cyclic mechanical loading on the fracture resistance of 3D-printed zirconia crowns in comparison to milled zirconia crowns.

**Materials and methods:**

Monolithic zirconia crowns (*n* = 30) were manufactured using subtractive milling (group M) and 3D additive printing (group P). Nine samples of each group were fractured under one-time loading while the other 6 samples were subjected to cyclic loading for 1.2 million cycles before being subjected to one-time loading until fracture. Scanning electron microscope (SEM) fractographic analysis was carried out on fractured fragments of representative samples.

**Results:**

The mean for fracture resistance of group M was 1890 N without cyclic loading and 1642 N after being subjected to cyclic loading, and they were significantly higher than that of group P (1658 N and 1224 N respectively).

**Conclusions:**

The fabrication technique and cyclic loading affect the fracture resistance of zirconia crowns. Although the fracture resistance values for the 3D-printed crowns were lower than those of the milled, still they are higher than the masticatory forces and thus could be considered being clinically acceptable.

**Clinical relevance:**

Concerning fracture resistance, 3D-printed crowns can withstand the masticatory forces for the long term without any cracks or failure.

## Introduction

Patients and dentists have been looking for metal-free tooth-colored restorations due to the increased esthetic demands, the more conservative preparations, and the high concerns of toxic and allergic reactions to certain alloys [1]. Yttria partially stabilized zirconia (Y-TZP) ceramics have been widely used because of their desirable esthetics, biocompatibility, superior fracture strength, and fracture toughness compared to other ceramics used in dentistry [2]. Core substructures can be fabricated, then veneered with glass ceramics in layering, press, or computer-aided design/computer-aided manufacturing (CAD/CAM) techniques. However, the veneer is directly exposed to chewing, clenching, and moisture, which might weaken it and result in cracking or chipping [3]. To overcome this problem, monolithic zirconia dental restorations with full anatomic contour have been developed without the need for adding a veneer layer [4].

The development of these materials and the CAD/CAM fabrication techniques has created a wide range of applications for dental restorations with conservative tooth preparation, such as partial coverage restorations, crowns, and bridges [5]. Monolithic zirconia dental restorations are usually fabricated by subtractive milling. This technique has some drawbacks such as the difficulty of milling a thin margin restoration without the possibility of chipping and the difficulty of fabricating a restoration with deep grooves and complex structures [6–8]. In addition, this technique produces a lot of material waste, deterioration of the burs, and high production costs [9].

The fast expansion and the development of materials and techniques used in additive manufacturing allowed the possibility of the production of monolithic zirconia dental restorations from a variety of materials; additive manufacturing techniques include selective laser sintering (SLS), selective laser melting (SLM), stereolithography (SLA), ink-jet printing (IJP), and fused deposition modeling (FDM) [10]. In this study, lithography-based ceramic manufacturing (LCM) technology was used for the fabrication of 3D-printed zirconia crowns because this is the only available and most advanced approach to fabricate a fully anatomical 3D-printed zirconia crown [11].

The fracture resistance of all-ceramic restorations is one of the major concerns in clinical applications of these materials, and it is influenced by many factors such as surface roughness, elastic modulus, crack resistance, and fabrication technique [12, 13]. Zirconia can resist crack propagation by transforming from tetragonal to monoclinic causing a slight increase in the volume of crystals and generating favorable compressive stresses around the crack in a mechanism called transformation toughening [14]. However, zirconia may undergo low-temperature degradation (LTD) in aqueous environment over time, which causes a reduction in its mechanical properties [15].

It has been well-established in the literature that fracture is the main cause of failure of dental ceramics after years of service inside the oral cavity, as they are subjected to repeated loads in the patient’s mouth, which can influence the long-term reliability of the crowns. Consequently, to estimate the mechanical performance of zirconia crowns, it is not sufficient to test them under one-time loading only, cyclic loading must be tested as well [16]. Several studies have reported a reduction in flexural stress and toughness of some dental ceramics after cyclic loading [17]. The reduction of mechanical strength due to cyclic load is caused by the propagation of cracks at existing defects in the microstructure [1]. Thus, the mechanical properties of the newly introduced 3D-printed zirconia restorations are very important to withstand the mastication forces and their long-term survival. However, the data available for their properties and long-term functions is limited and needs more investigation [18].

Accordingly, the aim of our study was to evaluate the fracture resistance of 3D-printed zirconia crowns and the effect of cyclic loading in an aqueous solution on their fracture resistance, compared to milled zirconia crowns. The first null hypothesis was that there would be no significant difference in the fracture resistance between the crowns fabricated by milling or 3D-printed techniques. The second null hypothesis postulated that the cyclic loading would not affect the fracture resistance of zirconia crowns fabricated by both techniques.

## Materials and methods

### Preparation criteria

An upper premolar typodont tooth was prepared using tapered stone with a round end to create a deep chamfer finish line with 1 mm thickness, axial wall convergence of 6%, and 1.5 mm occlusal reduction [19, 20].

Thirty impression replicas were taken for the prepared tooth using addition silicone impression material (Elite HD + , Zhermack, Rovigo, Italy) and putty soft and light consistency to pour 30 epoxy stumps (Kemapoxy 150 3D, CMB, Egypt) that were allowed to set until full hardening [21].

### Scanning designing and fabrication of zirconia crowns

After scanning the epoxy stump with an extraoral scanner (Medit T500, Medit, Seoul, Korea), the crowns were digitally designed using exocad software (version 3.0, Darmstadt, Germany).

A total of 30 zirconia crowns (3 mol % yttria-stabilized zirconia) were fabricated using 2 different fabrication techniques with 15 crowns in each group. As a control group (group M), IPS e.max ZirCAD LT (Ivoclar Vivadent, New York, USA) zirconia crowns were milled using a milling machine (DGSHAPE DWX-520 milling machine, Roland Company, Willich, Germany); then, they were sintered in a zirconia furnace (Tabeo, MIHM VOGT, Stutensee Blankenloch, Germany) up to 1530 °C, while in group P, Lithoz 210 3Y (Lithoz GmbH, Vienna, Austria) zirconia crowns were 3D printed using a CeraFab7500 printer (Lithoz GmbH, Vienna, Austria); then, they were preconditioned up to 120 °C for 134 h, then debound at up to 1000 °C for 103 h, then sintered at up to 1450 °C for 17 h in 3 consecutive furnaces (Nabertherm oven, Nabertherm GmbH, Lilienthal, Germany). The materials used for each group in the study and the sintering parameters are listed in Tables 1 and 2.

**Table 1:** Materials used for each group in the study

Group name	Product name	Manufacturer	Composition
Milled group (group M)	IPS e.max ZirCAD LT, Ivoclar Vivadent, USA LOT: Y43302	Ivoclar Vivadent AG, USA	Zirconium oxide (ZrO_2_) 88.0–95.5 wt%, yttrium oxide (Y_2_O_3_) > 4.5– ≤ 6.0 wt%, hafnium oxide (HfO_2_) ≤ 5.0 wt%, aluminum oxide (Al_2_O_3_) ≤ 1.0 wt%, other oxides for coloring ≤ 1.0 wt%
Printed group (group P)	Lithacon 3Y 210, Lithoz, Vienna, Austria LOT: AB0722038	Lithoz GmbH, Vienna, Austria	Zirconium oxide (ZrO_2_) 3 mol-%Y_2_O_3_ stabilized

**Table 2:** Firing parameters of zirconia crowns

Group		Furnace	Highest temp in °C	Cycle time
M	Sintering	Tabeo, MIHM VOGT, Stutensee Blankenloch, Germany	1530	400 min (6 h and 40 min)
P	Preconditioning	Nabertherm TR 60, Nabertherm GmbH, Lilienthal, Germany	120	134 h
	Debinding	Nabertherm L 40/11 BO, Nabertherm GmbH, Lilienthal, Germany	1000	103 h
	Sintering	Nabertherm HT 40/17, Nabertherm GmbH, Lilienthal, Germany	1450	17 h

A power analysis study was performed using the G power statistical power analysis program (version 3.1.9.4, University of Düsseldorf, Germany) to estimate the sample size. A total sample size of 30 samples (15 in each group, 9 to be used for measuring fracture without fatigue and 6 for measuring fracture after cyclic fatigue) was found to be sufficient to detect a large effect size (*d*) ranging from 1.46 to 1.82, with an actual power (1 − *β* error) of 0.8 (80%) and a significance level (*α* error) of 0.05 (5%) for a two-sided hypothesis test [22].

**Fig. 1:**
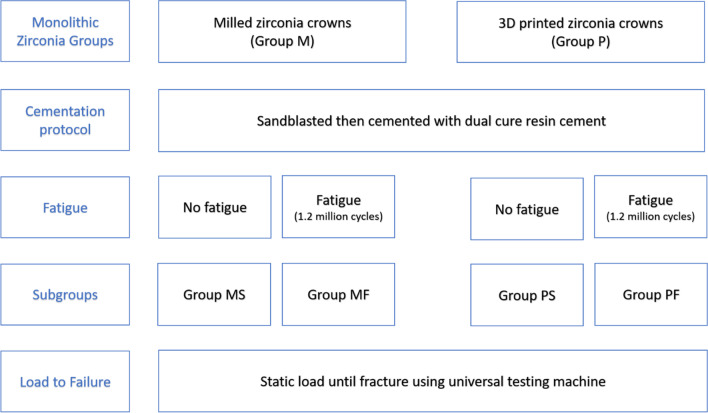
Schematic diagram showing the study groups

The study design and grouping are illustrated in Fig. 1.

### Crown cementation

A sharp dental explorer was used to check for proper seating of the crowns on the corresponding epoxy dies before cementation. The fitting surface of the crowns was sandblasted using 50-µm alumina particles at 2 bar for 10 s at a distance of 10 mm [23], while no surface finish was made to the outer surface after fabrication. A universal dual-cured resin luting cement (Duo-Link, Bisco, Schaumburg, USA) was auto-mixed and injected into the crown fitting surface; then, the crowns were seated on the epoxy dies. Initial light curing was made for 3 s; then, the excess cement was removed using a sharp dental explorer followed by curing for 20 s on each surface.

### One-time loading and cyclic loading fracture resistance testing

**Fig. 2:**
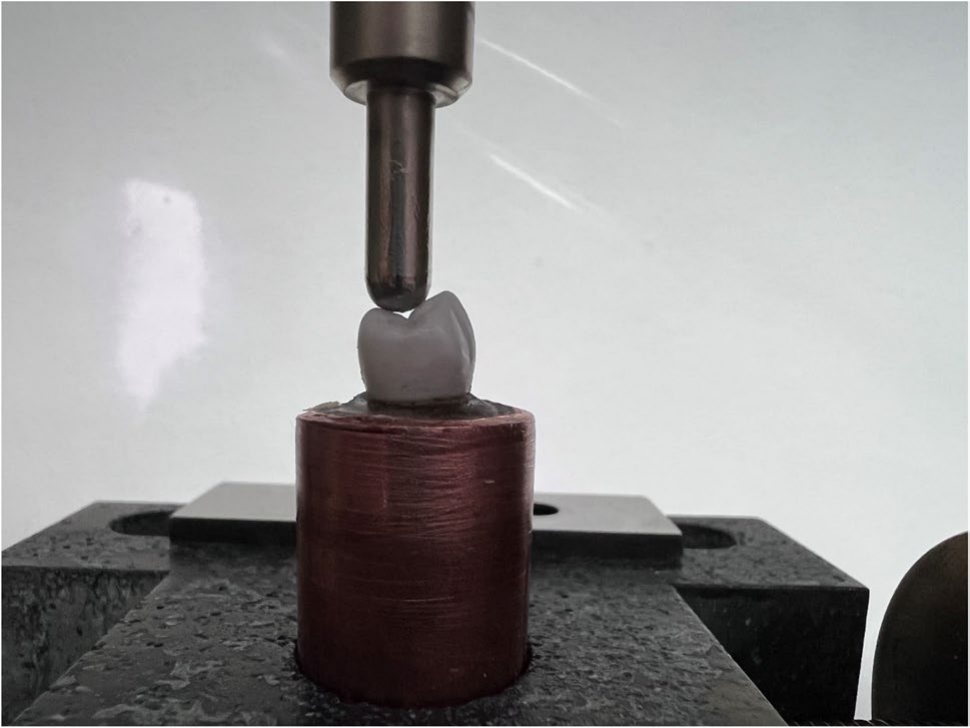
Load to fracture test using the universal testing machine. The piston with a tip diameter of 5 mm is applied perpendicular to the occlusal surface and driven at a crosshead speed of 1 mm/min

After the cementation of the dental crowns, the epoxy dies were trimmed to fit centrally in copper tubes and fixed with auto-polymerized resin (Technovit 4004, Kulzer GmbH, Hanau, Germany). Samples of each group (*n* = 9, MS, PS) were loaded using a metal stylus with a 5-mm-diameter spherical tip with a speed of 1 mm/min which was applied directly to the center of each crown and perpendicular to the occlusal surface until fracture using a universal testing machine (Zwick Zmart-Pro, ZwickRoell GmbH & Co. KG, Ulm, Germany) (see Fig. 2).

**Fig. 3:**
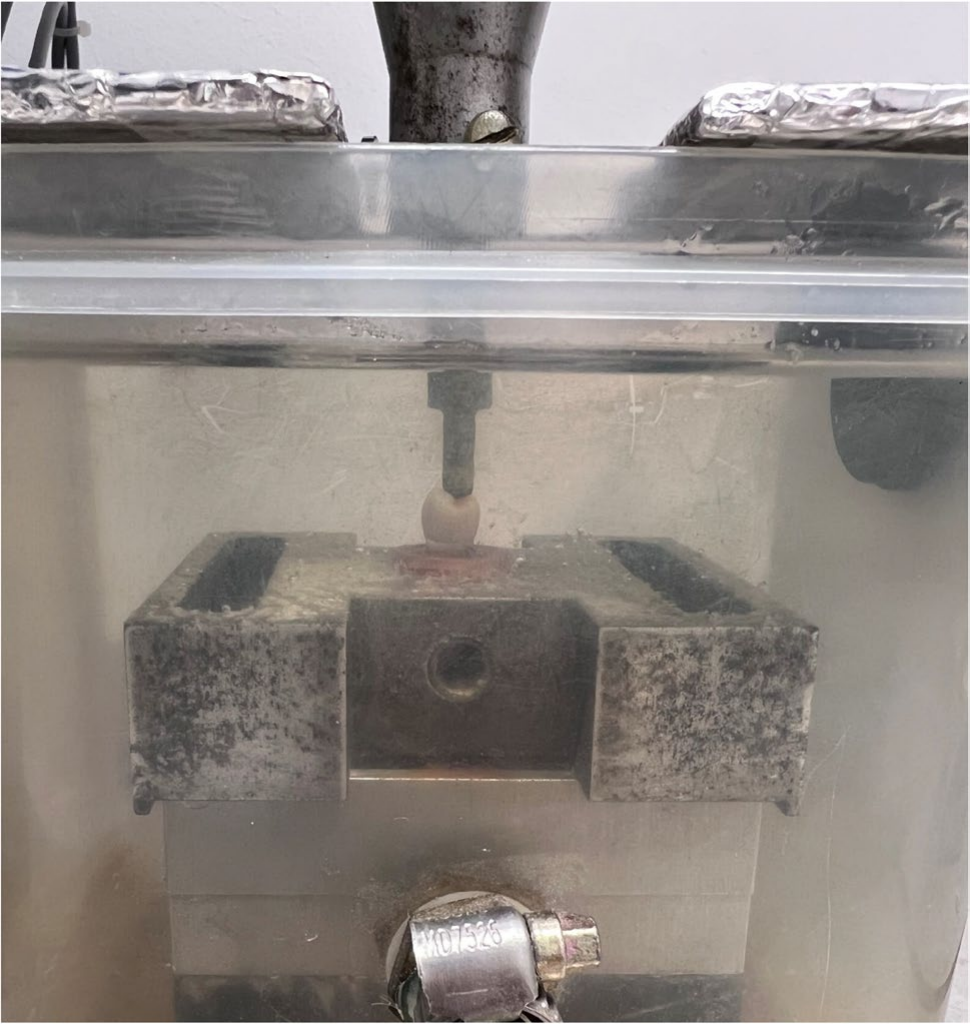
The basin used to realize the wet environment with a temperature of 37 ± 2 °C

The other 6 samples of each group (MF, PF) were subjected to dynamic mechanical loading using a commercial pneumatic setup (“Dyna-Mess TP 5kN HF”, DYNA-MESS Prüfsysteme GmbH, Stolberg, Germany). 1.2 million cycles were applied with a load between 20 and 200 N at 2 Hz in distilled water 37 ± 2 °C to simulate approximately 5 clinical years [18, 24, 25]. Specimens were fixed in a specimen holder positioned so that the forces could act directly on the center and perpendicular to the occlusal surface using a metal stylus with a 5-mm-diameter spherical tip in a small basin connected by a temperature-controlled reservoir of purified water as shown in Fig. 3. The tempered purified water was constantly pumped from the reservoir to the basin and overflowing fluid flows back through a second flexible tube. Specimens were examined using a stereomicroscope (Wild Leica M8, Heerbrugg, Switzerland) equipped with a digital camera (Leica DFC 420 C, Leica Mikrosysteme, Wetzlar, Germany) at × 25 magnification. Samples with any cracks or defects were discarded. All specimens subjected to dynamic loading were free from any cracks, and then, they were loaded until fracture using the previously mentioned method.

### Fractographic analysis

Representative fractured crown samples from each group were selected, cleaned using alcohol in an ultrasonic cleaner, and coated with a thin gold/platinum layer using a sputter coater (Scancoat Six, Edwards High Vacuum, England, UK) for 60 s. After sputtering, the samples were ready to be examined under a scanning electron microscope (SEM, Philips XL 30 CP, Philips, Eindhoven, Netherland) at an operating voltage of 10 kV and a spot size of 5 using secondary emission modes.

### Statistical analysis

Numerical data were represented as mean with a 95% confidence interval, standard deviation (SD), and minimum and maximum values. Shapiro–Wilk’s test was used to test for normality. The homogeneity of variances was tested using Levene’s test. Data showed parametric distribution and variance homogeneity and were analyzed using a two-way ANOVA test. The significance level was set at *p* < 0.05 within all tests. Statistical analysis was performed with R statistical analysis software (R: A language and environment for statistical computing. R Foundation for Statistical Computing, Vienna, Austria. URL https://www.R-project.org/…) version 4.1.3 for Windows (R Core Team 2022).

## Results

**Fig. 4:**
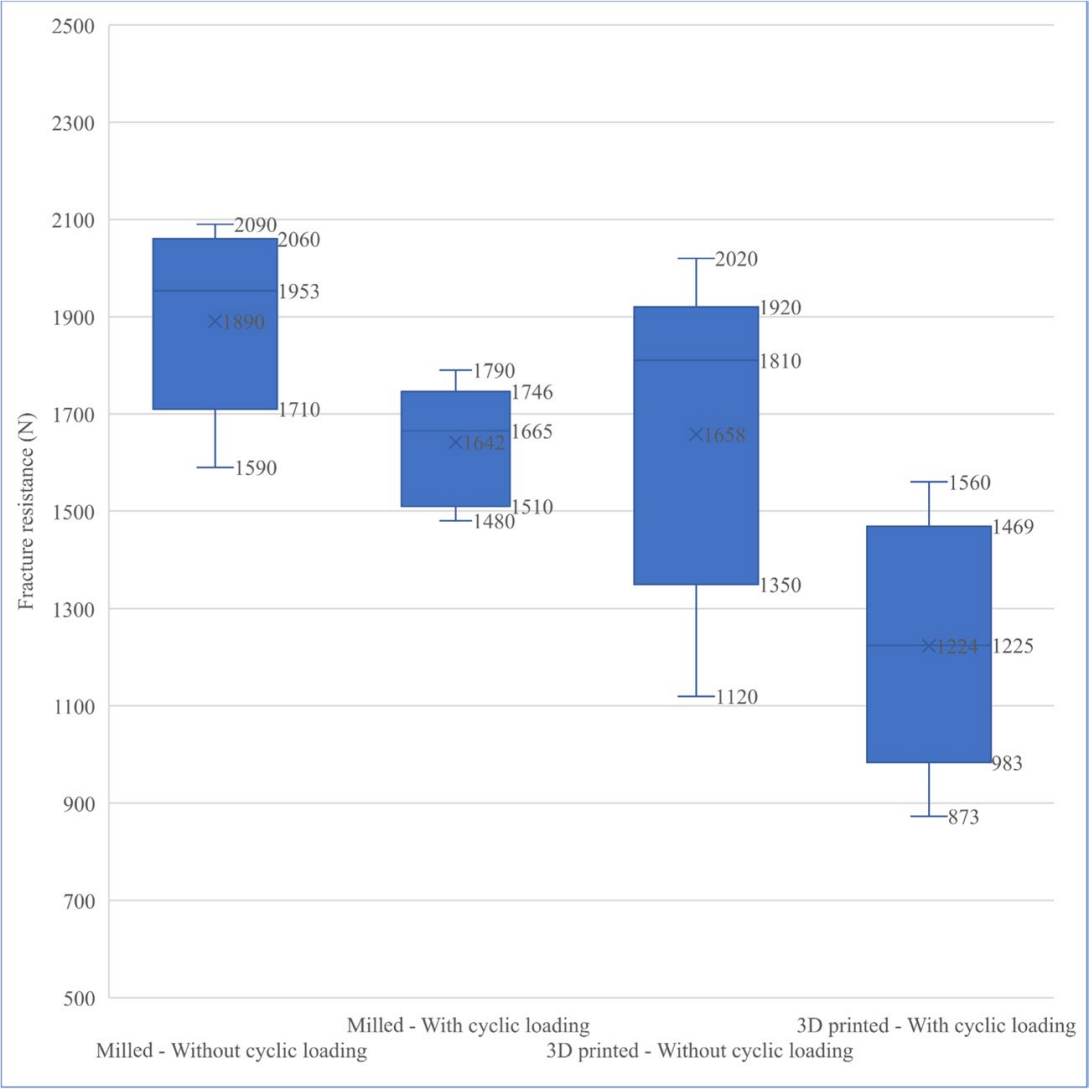
Box plot showing fracture resistance values in N

Descriptive statistics for fracture resistance values are presented in Fig. 4. It showed that the mean for the fracture resistance of group MS was 1890 ± 191 N and it decreased to 1642 ± 127 N after being subjected to dynamic mechanical loading in group MF while the fracture resistance of group PS was 1658 ± 333 N and decreased after dynamic mechanical loading to 1224 ± 263 N in group PF. Results of two-way ANOVA for the effect of tested variables on fracture resistance are presented in Table 3. It was found that the fabrication technique (*p* = 0.002) and the mechanical cycling loading (*p* = 0.001) had a significant effect on fracture resistance while there was no significant interaction between tested variables (*p* = 0.325).

**Table 3:** Two-ANOVA for fracture resistance, values in bold indicate significant difference

Parameter	Sum of squares	*df*	Mean square	*f-*value	*p-*value
Fabrication technique	705,964	1	705,964	11.4	0.002*
Cyclic loading	838,914	1	838,914	13.6	0.001*
Technique * loading	62,163	1	62,163	1	0.325
Error	1,606,803	26	61,800		

^*^Significant (*p* < 0.05)

**Fig. 5:**
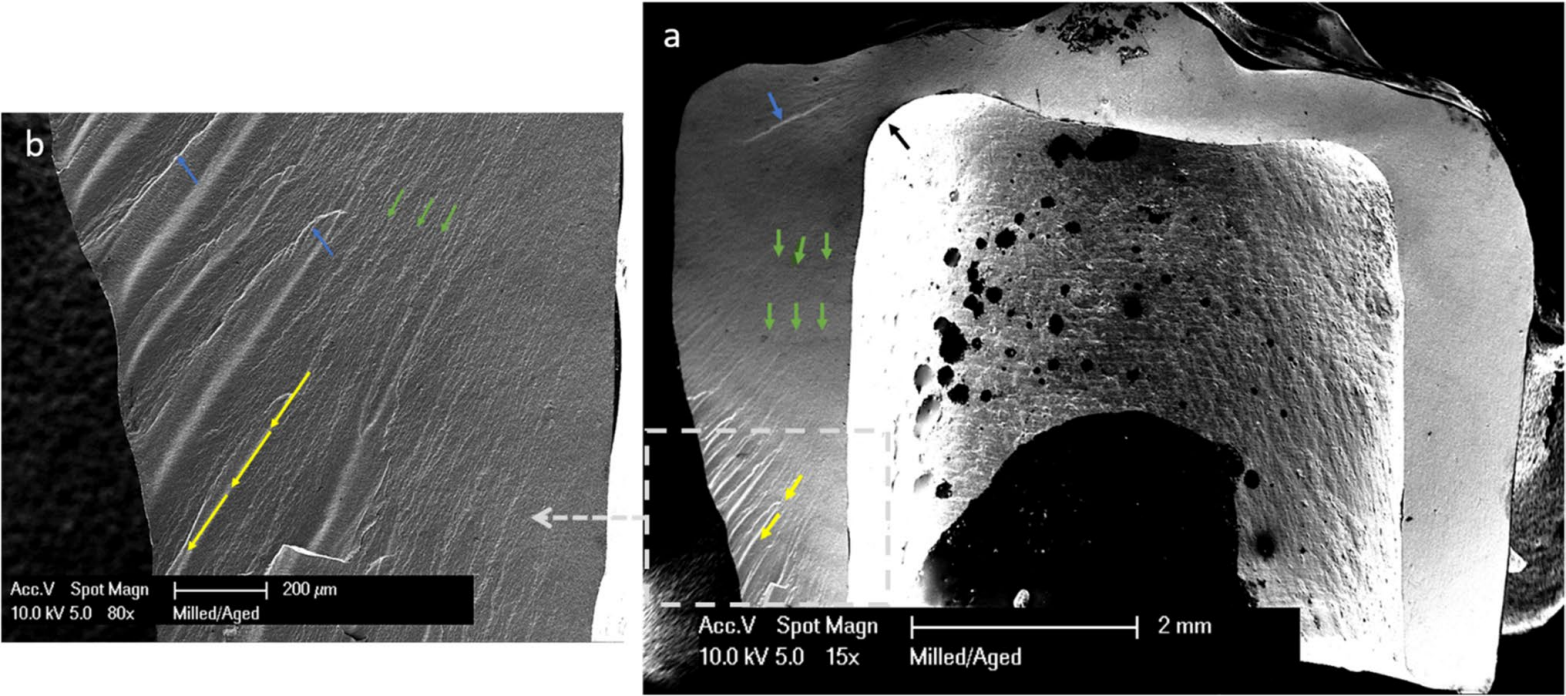
A fractographic map of milled zirconia after dynamic loading. **a** SEM magnified at 15 ×. **b** Detailed image magnified at 80 ×. Black arrows show crack origin. Blue arrows show twist hackles. Green arrows show arrest lines. Yellow lines show the direction of crack propagation from the inner surface of the crowns and move outwards

**Fig. 6:**
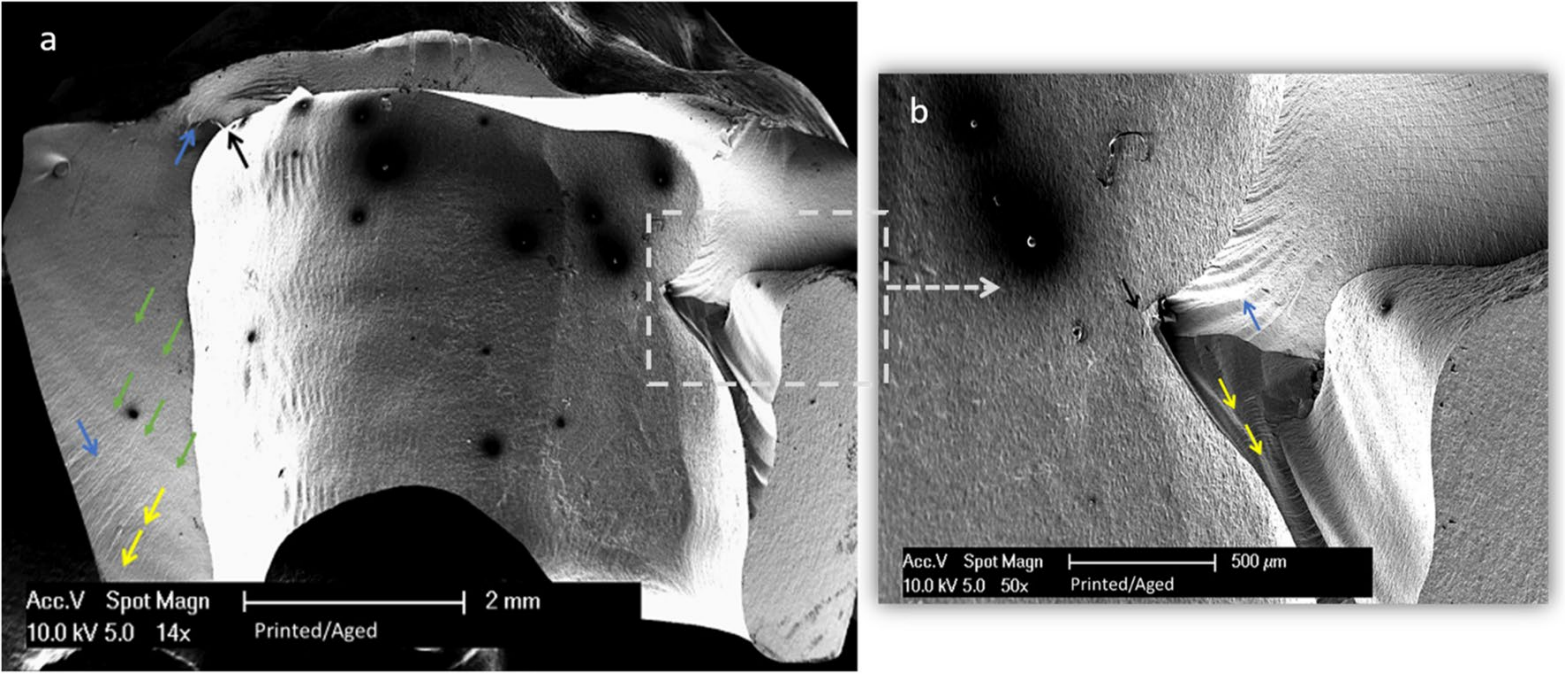
A fractographic map of 3D-printed zirconia after dynamic loading. **a** SEM magnified at 14 ×. **b** Detailed image magnified at  50 ×. Black arrows show crack origin. Blue arrows show twist hackles. Green arrows show arrest lines. Yellow lines show the direction of crack propagation from the inner surface of the crowns and move outwards

SEM showed similar fracture patterns in the selected samples, the cracks in all the groups originated away from the point of load application, and the direction of crack propagation started from the tensile zone at the internal surface and propagated outwards to the external surface of the crown. Twist hackles and arrest lines are present as shown in Figs. 5 and 6.

## Discussion

The first null hypothesis of the present study that there would be no significant difference in the fracture resistance between the crowns fabricated by milling and 3D-printing techniques was rejected. Also, the second null hypothesis was rejected, as the cyclic loading decreased the fracture resistance of zirconia crowns fabricated by both techniques.

In the present study, premolars were used as they require an esthetic restoration that can withstand high masticatory forces [19, 20]. Similar stumps and crowns were used for standardization. Epoxy resin was used to prepare the stumps as it has an elastic modulus of 11.8 GPa, which is close to that of dentin (18.6 GPa) [21, 26]. In this regard, the elastic modulus of the stump has a significant effect on the fracture resistance of all-ceramic crowns [27], as it was reported that increasing the elastic modulus of the stump material leads to an increase in the fracture resistance of all-ceramic crowns [28]. Many studies have evaluated the fracture strength of all ceramic crowns using stainless steel [27], copper [29], acrylic resin [30], epoxy resin [31, 32], and dentin [33]. According to Yucel et al. [21], the stress distributions of dentin and epoxy resin stump could simulate the clinical conditions with realistic fracture strength values compared to those of stainless steel and copper.

Accelerated aging tests provide essential information on lifetime predictions of ceramic restorations; the applied fatigue protocol with 1.2 million cycles is equivalent to 5 years of clinical service for dental restorations [34]. The cyclic loading was done at a frequency of 2 Hz as the range of the chewing activity is between 0.94 nd 2.17 Hz [35].

In this study, the immediate fracture resistance of all crowns (1890 ± 191 N for the milled crowns and 1658 ± 333 for the 3D-printed crowns) exceeded 790 N, which is higher than the maximum biting forces (450–520 N) that increases to 790 N in bruxism [36, 37]. After cyclic loading, all the specimens showed no cracks or catastrophic failure which is similar to other studies that tested zirconia crowns [34, 38]. The fracture resistance of the crowns was reduced after cyclic loading (1658 N for the milled crowns and 1224 N for the 3D-printed crowns); however, it was higher than the maximum biting forces. Accordingly, crowns fabricated by both milling and 3D-printing techniques could withstand up to 5 years of clinical service in the oral cavity.

Milled zirconia crowns showed significantly higher fracture resistance than the 3D-printed zirconia crowns. These results were consistent with the results of Zhai et al. [39].

This may be contributed to the higher amount of porosity in the 3D-printed zirconia crowns. This effect has been widely studied by Branco et al. [40], who found no features of interlayer delamination; thus, they are not related to a bad interlayer binding. They also stated that the mechanical properties of the zirconia ceramics are affected by porosity and density; they found that the porosities in the 3D-printed zirconia were randomly distributed and they attributed the presence of porosities to the entrapment of air bubbles in the paste of the 3D-printed zirconia.

Fractographic analysis showed similar fracture patterns for the examined specimens of all the tested crowns. The fracture origin was located away from the loading site, in the tensile zone (inner surface of the crowns), and the direction of crack propagation was outwards; this could be explained by the transverse expansion that occurs in the abutment material owing to the Poisson effect when the crown was subjected to occlusal loading. The Poisson ratio of epoxy (0.31 μ) is similar to that of dentin (0.3 μ) [21]; therefore, the degree of bulging of the epoxy model was expected to be similar to dentin during mastication when subjected to the same occlusal load. The bulging causes the development of hoop stress that causes the fracture [41]. They also showed twist hackles which are formed due to a new stress direction, having the appearance of lances where the small hackle lines merge, as well as arrest lines.

Among the limitations of this study is the use of abutments with no mobility; the dynamic load was vertical with no chewing cycles and in the clinical situation; the crown’s design may vary according to the patient. Additionally, the effects of other environmental factors, such as saliva, or the effects of different kinds of beverages and pH fluctuation were not taken into consideration in the present study.

## Conclusion

Among the limitations of this in-vitro study, it could be concluded that

1. Zirconia crowns fabricated by the 3D-printing technique can withstand chewing forces for over 4 years (1.2 million cycles).
2. The fracture resistance of these crowns will decrease during function in the oral cavity but is still higher than the masticatory forces (450–520 N).
3. The fracture resistance of the printed crowns is lower than that of the milled crowns but is still higher than the masticatory forces (450–520 N).

## Data Availability

Data available on request from the authors.
